# Galectin-1 knockdown improves drug sensitivity of breast cancer by reducing P-glycoprotein expression through inhibiting the Raf-1/AP-1 signaling pathway

**DOI:** 10.18632/oncotarget.15341

**Published:** 2017-02-15

**Authors:** Fang Wang, Pengwei Lv, Yuanting Gu, Lin Li, Xin Ge, Guangcheng Guo

**Affiliations:** ^1^ Department of Breast Surgery, The First Affiliated Hospital of Zhengzhou University, 450000, China

**Keywords:** Galectin-1, multidrug resistance, P-glycoprotein, Raf-1/AP-1, breast cancer

## Abstract

Galectin-1 (Gal-1), a member of the galectin family of carbohydrate binding proteins, plays a pivotal role in various cellular processes of tumorigenesis. The regulatory effect of Gal-1 on multidrug resistance (MDR) breast cancer cells is still unclear. qRT-PCR and western blot showed that Gal-1 and MDR gene 1 (*MDR1*) were both highly expressed in breast tumor tissues and cell lines. MTT assay and flow cytometry revealed that Gal-1 knockdown improved sensitivity to paclitaxel (PTX) and adriamycin (ADR) in MCF-7/PTX and MCF-7/ADR cells via inhibition of cell viability and promotion of cell apoptosis, while MDR1 overexpression weakened the sensitivity to PTX and ADR induced by Gal-1 knockdown. Furthermore, the negative effects of Gal-1 knockdown on sensitivity to PTX and ADR in MCF-7/PTX and MCF-7/ADR cells were revealed to be mediated via the suppression of Raf-1/AP-1 pathway. In conclusion, Gal-1 knockdown dramatically improved drug sensitivity of breast cancer by reducing P-glycoprotein (P-gp) expression via inhibiting the Raf-1/AP-1 pathway, providing a novel therapeutic target to overcome MDR in breast cancer.

## INTRODUCTION

Breast cancer is one of the most prevalent cancers and the gradually increasing incidence of breast cancer is the leading cause of cancer death among women in the world [1]. Chemotherapy is the main treatment of breast cancer. Chemotherapeutic agents such as paclitaxel (PTX) and adriamycin (ADR) have been widely used in the treatment of various solid tumors including breast cancer [2]. However, multidrug resistance (MDR) gradually emerges in clinical trials by using chemotherapeutic agents alone or combined with other antineoplastic agents and is becoming a major clinical obstacle and paramount factor in the failure of breast cancer chemotherapy and the human body has become insensitive to antitumor drugs due to MDR [3].

The mechanisms related to MDR are multifarious, such as the increased drug efflux, activation of DNA repair process and the deregulation of drug-induced apoptosis and the major factor of MDR pathogenesis is the high expression of MDR proteins of adenosine triphosphate (ATP)-binding cassette (ABC) transporters in cell membranes, especially multidrug efflux transporter P-glycoprotein (P-gp) [4, 5]. P-gp, a plasma membrane glycoprotein encoded by the ATP-binding cassette sub-family B member 1 (ABCB1, also known as multidrug resistance gene 1 (MDR1)), is one of the main multidrug transporters and has an important impact on the absorption, distribution and elimination of chemotherapeutic drugs [6]. P-gp can recognize chemotherapeutic drugs as substrates, pump substrates out of tumor cells and restrict the uptake of substrates in an ATP-dependent manner [7], which leads to a decrease of intracellular drug accumulation and soon causes resistance to numerous drugs in cancer cells [8]. Therefore, P-gp is regarded as the most important MDR-regulatory drug target and inhibition of P-gp expression would be an effective therapeutic approach to overcome MDR and enhance the therapeutic effect.

Galectin-1 (Gal-1), a homodimer of 14 kDa subunits, is a member of the galectin family of carbohydrate binding proteins and contains two β-galactoside binding sites [9]. Gal-1 plays a pivotal role in various cellular processes of tumorigenesis, such as cell proliferation, apoptosis, cell invasiveness, migration, and angiogenesis and regulates the interaction between tumor cells and components of tumor microenvironment [10]. Besides, Gal-1 has been demonstrated as a therapeutic target by identifying the overexpression of Gal-1 in human cancer including breast cancer [10, 11].

Raf, a member of Raf family of serine/threonine kinases, is ubiquitously expressed and activated by the Ras family of GTPases, which is involved in survival, cell growth and differentiation of tumors [12]. Raf is involved in a broad range of cancers and functions as an oncogene that regulates mitogen-activated protein (MAPK) pathway and cell proliferation to promote tumourigenesis [13]. The activation of MAPK pathway, which is regulated by phosphorylation of some signaling protein kinases including Ras, Raf, and Raf upstream activator PKC, has important roles in mediating signals caused by growth factors, cytokines, and environment stress and is associated with cell proliferation, survival, differentiation and apoptosis [14]. It has been reported that an intact Gal-1 interface of stacked dimers of H-Ras, Raf and Gal-1 as building blocks is required for Gal-1 to modulate Ras nanoclustering, thus representing a potential drug target site in cancer associated Ras-mutations [15]. The proto-oncogenes c-Jun and c-Fos are the main components of the AP-1 transcription factor complex in the downstream of Ras/Raf and are crucial for Ras/Raf-mediated cell proliferation and transformation [16]. Additionally, AP-1 regulates the activation of numerous genes transcription and is a main target of MAPK signaling pathway [17]. AP-1 is associated with various cellular processes, such as cell differentiation, proliferation, survival and apoptosis [18].

In this study, we aimed to investigate the effect of Gal-1 knockdown on cell proliferation and apoptosis of the MDR breast cancer cell lines and the relationship between Gal-1 and Raf-1/AP-1 signaling pathway to further study the molecular mechanism of MDR, providing a novel therapeutic approach to overcome MDR in breast cancer.

## RESULTS

### Gal-1 and MDR1 levels were both upregulated in breast tumor tissues and cell lines

**Figure 1:**
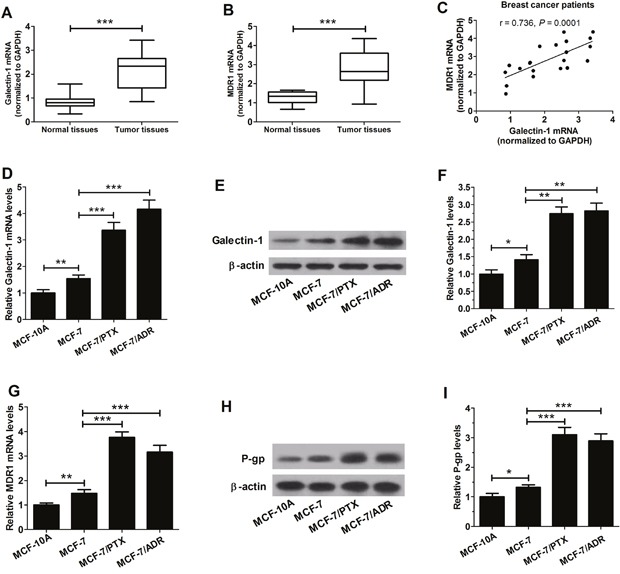
Expression of Gal-1 and MDR1 in breast tumor tissues and cells lines **A**. qRT-PCR was used to measure the level of Gal-1 mRNA (A) and MDR1 mRNA **B**. in breast tumor tissues and normal tissues. **C**. A positive correlation between MDR1 and Gal-1 mRNA expression. **D**. The mRNA expressions of Gal-1 in normal breast cell line (MCF-10A) and breast tumor cell lines (MCF-7, MCF-7/PTX and MCF-7/ADR) cells were detected by qRT-PCR. **E**. and **F**. The levels of Gal-1 protein in MCF-10A, MCF-7, MCF-7/PTX and MCF-7/ADR cells were evaluated by western blot. **G**. The mRNA expressions of MDR1 in MCF-10A, MCF-7, MCF-7/PTX and MCF-7/ADR cells were determined by qRT-PCR. **H**. and **I**. The levels of P-gp protein in MCF-10A, MCF-7, MCF-7/PTX and MCF-7/ADR cells were detected by western blot. Data are shown as mean ± SD. *P < 0.05, **P < 0.01.

To evaluate the potential role of Gal-1 and MDR1 in breast cancer, the expression levels of Gal-1 and MDR1 in MCF-10A, MCF-7, MCF-7/PTX, MCF-7/ADR cells, normal tissues and breast tumor tissues samples were detected by qRT-CPR and western blot. As shown in Figure 1A and 1B, qRT-PCR results indicated that Gal and MDR1 were significantly upregulated in breast tumor tissues compared with that in normal tissues. In addition, the mRNA expressions of Gal-1 in breast cancer patients were positively correlated with MDR1 (*r* = 0.736, *P* = 0.0001) (Figure 1C). Besides, Gal-1 was found to be dramatically higher in mRNA and protein levels in MCF-7 cells than that in MCF-10A cells and the mRNA and protein levels of Gal-1 in MCF-7/PTX and MCF-7/ADR cells were strikingly higher than that in MCF-7 cells (Figure 1D, 1E and 1F). The expressions of MDR1 mRNA and protein P-gp in MCF-7 cells were significantly higher than that in MCF-10A cells and dramatically lower than that in MCF-7/PTX and MCF-7/ADR cells (Figure 1G, 1H and 1I). These differential expressions suggested that Gal-1 and MDR1 may play important roles in breast cancer development and resistance.

### Gal-1 knockdown enhanced sensitivity to PTX and ADR in MCF-7/PTX and MCF-7/ADR cells

**Figure 2:**
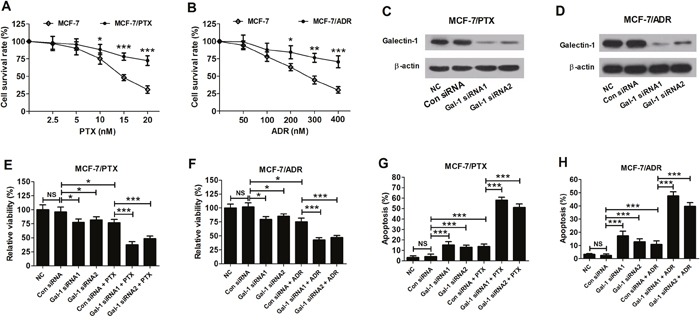
Effects of Gal-1 knockdown on sensitivity to PTX and ADR in MCF-7/PTX and MCF-7/ADR cells **A**. Cell survival rates in MCF-7 and MCF-7/PTX with a series of PTX concentrations (2.5, 5, 10, 15, and 20 nM) treatment were detected by MTT assay. **B**. Cell survival rates in MCF-7 and MCF-7/ADR with a series of ADR concentrations (50, 100, 200, 300, and 400 nM) treatment were detected by MTT assay. Western blot was used to detect the levels of Gal-1 in MCF-7/PTX cells **C**. and MCF-7/ADR cells **D**. transfected with Gal-1 siRNA1, Gal-1 siRNA2 or si-control. **E**. and **G**. The relative cell viability and cell apoptosis in MCF-7/PTX cells with Gal-1 siRNA1 or Gal-1 siRNA2 transfection or together with 15 nM PTX treatment was determined by MTT assay and flow cytometry. **F**. and **H**. The relative cell viability and cell apoptosis in MCF-7/ADR cells with Gal-1 siRNA1 or Gal-1 siRNA2 transfection or together with 300 nM ADR treatment was determined by MTT assay and flow cytometry. Data are presented as mean ± SD. *P < 0.05, **P < 0.01, ***P < 0.001.

To determine cell sensitivity to PTX and ADR, MCF-7, MCF-7/PTX and MCF-7/ADR cells were treated with different concentrations of PTX or ADR for 24 h. Cell survival rates were assessed by MTT assay and the results showed that the cell survival rates of MCF-7/PTX cells at 10 nM, 15 nM and 20 nM of PTX and MCF-7/ADR cells at 200 nM, 300 nM and 400 nM of ADR were significantly higher than those in MCF-7 cells (Figure 2A and 2B), which confirmed the PTX resistance in MCF-7/PTX cells and ADR resistance in MCF-7/ADR cells. IC50 of MCF cells to PTX (15 nM) and IC50 of MCF cells to ADR (300 nM) were chosen for sequential studies.

Considering the upregulation of Gal-1 in MCF-7/PTX and MCF-7/ADR cells, Gal-1 knockdown was performed to observe its effect on the drug resistance of MCF-7/PTX and MCF-7/ADR cells. The cell viability and apoptosis in MCF-7/PTX and MCF-7/ADR cells transfected with Gal-1 siRNA1 or Gal-1 siRNA2 were evaluated by MTT assay and flow cytometry with or without PTX or ADR treatment. As presented in Figure 2C and 2D, Gal-1 siRNA1 and Gal-1 siRNA2 significantly decreased the levels of Gal-1 in MCF-7/PTX and MCF-7/ADR cells, indicating the efficiency of siRNAs in silencing Gal-1 expression. Gal-1 knockdown or (PTX or ADR) treatment resulted in an obvious decrease in cell viability; however, combination of Gal-1 knockdown with PTX or ADR resulted in a lower cell viability than chemotherapy group alone in MCF-7/PTX and MCF-7/ADR cells (Figure 2E and 2F). Gal-1 knockdown or (PTX or ADR) treatment led to a marked increase in cell apoptosis; however, combination of Gal-1 knockdown with PTX or ADR induced a higher cell apoptosis than chemotherapy group alone in MCF-7/PTX and MCF-7/ADR cells (Figure 2G and 2H). These results showed that Gal-1 knockdown improved the sensitivity against PTX and ADR in MCF-7/PTX and MCF-7/ADR cells via the inhibition of cell viability and induction of cell apoptosis.

### Overexpression of MDR1 reduced the sensitivity to PTX and ADR induced by Gal-1 knockdown in MCF-7/PTX and MCF-7/ADR cells

**Figure 3:**
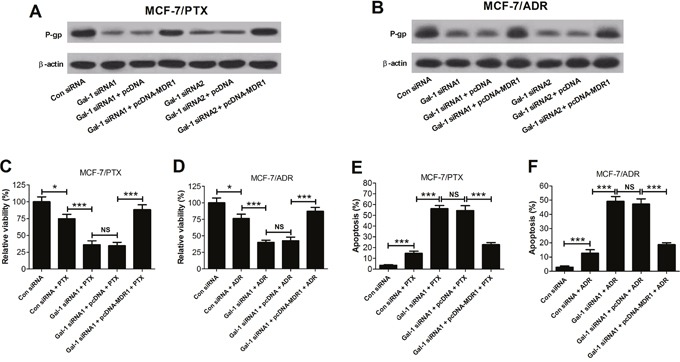
Effects of MDR1 overexpression on the sensitivity to PTX and ADR induced by Gal-1 knockdown in MCF-7/PTX and MCF-7/ADR cells The levels of P-gp in MCF-7/PTX cells **A**. and MCF-7/ADR cells **B**. cotransfected Gal-1 siRNAs or (Gal-1 siRNAs + pcDNA-MDR1) were detected by western blot. **C**. and **E**. With 15 nM PTX treatment, the relative cell viability and cell apoptosis in MCF-7/PTX cells cotransfected Gal-1 siRNA1 with pcDNA 3.1-MDR1 were evaluated by MTT assay and flow cytometry. **D**. and **F**. With 300 nM ADR treatment, the relative cell viability and cell apoptosis in MCF-7/ADR cells cotransfected Gal-1 siRNA1 with pcDNA 3.1-MDR1 were evaluated by MTT assay and flow cytometry. Data are presented as mean ± SD. *P < 0.05, ***P < 0.001.

To explore the effect of Gal-1 knockdown or MDR1 overexpression on P-gp protein in MCF-7/PTX and MCF-7/ADR cells, cells were transfected Gal-1 siRNA1, Gal-1 siRNA2, (Gal-1 siRNA1 + pcDNA-MDR1) or (Gal-1 siRNA2 + pcDNA-MDR1). The western blot results indicated that Gal-1 siRNA1 and Gal-1 siRNA2 both significantly decreased the levels of P-gp in MCF-7/PTX and MCF-7/ADR cells compared with corresponding control groups, while MDR1 overexpression alleviated the inhibition effects of Gal-1 siRNA1 or Gal-1 siRNA2 on P-gp (Figure 3A and 3B).

To explore the effect of overexpression of MDR1ondrug sensitivity induced by Gal-1 knockdown in MCF-7/PTX and MCF-7/ADR cells, cells were treated with (Gal-1 siRNA1 + drug) or (Gal-1 siRNA1 + drug + pcDNA-MDR1). The MTT assay showed that combination of Gal-1 siRNA1 and PTX or Gal-1 siRNA1 and ADR strikingly suppressed the relative cell viability of MCF-7/PTX and MCF-7/ADR cells compared to (Con siRNA + drug) group, whereas MDR1 overexpression reversed these negative effects (Figure 3C and 3D). Flow cytometry assay indicated that Gal-1 siRNA1 and PTX or Gal-1 siRNA1 and ADR obviously induced apoptosis of MCF-7/PTX and MCF-7/ADR cells compared with (Con siRNA + drug) group, while MDR1 overexpression attenuated the induction effects (Figure 3E and 3F). These data demonstrated that overexpression of MDR1 significantly decreased the sentivity to PTX and ADR induced by Gal-1 knockdown in MCF-7/PTX and MCF-7/ADR cells via the increase of cell viability and reduction of cell apoptosis.

### Gal-1 knockdown significantly suppressed the Raf-1/AP-1 signaling pathway

**Figure 4:**
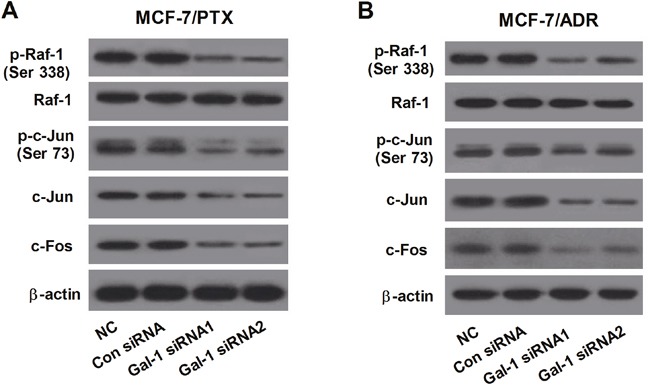
Gal-1 knockdown significantly suppressed the Raf-1/AP-1 signaling pathway The levels of p-Raf-1 (Ser338), Raf-1, p-c-Jun (Ser73), c-Jun and c-Fos in MCF-7/PTX cells **A**. and MCF-7/ADR cells **B**. transfected with Gal-1 siRNA1, Gal-1 siRNA2 or Con siRNA were determined by western blot β-actin was used for normalization.

The levels of p-Raf-1 (Ser338), Raf-1, p-c-Jun (Ser73), c-Jun and c-Fos were determined by western blot to investigate the effect of Gal-1 knockdown on the Raf-1/AP-1 signaling pathway. As compared with Con siRNA group, Gal-1 siRNA1 and Gal-1 siRNA2 significantly decreased the levels of p-Raf-1 (Ser338), p-c-Jun (Ser73), c-Jun and c-Fos in MCF-7/PTX and MCF-7/ADR cells, indicating that Gal-1 knockdown dramatically decreased the phosphorylation of Raf-1 and the induction of AP-1 activity in MCF-7/PTX and MCF-7/ADR cells (Figure 4A and 4B).

### Galectin-1 knockdown enhanced sensitivity to PTX and ADR by reducing P-glycoprotein expression through inhibiting the Raf-1/AP-1 signaling pathway

**Figure 5:**
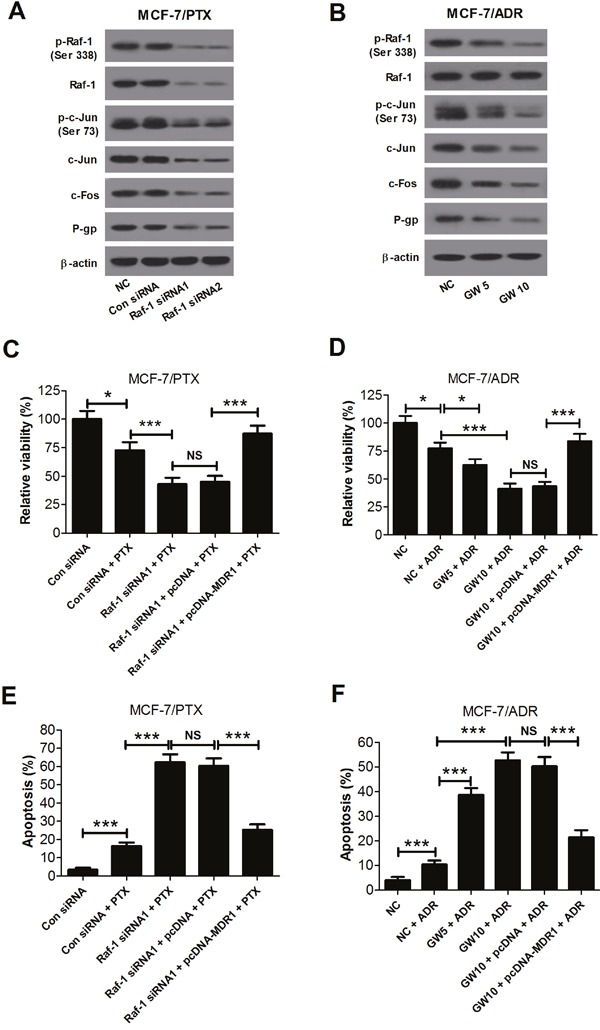
Gal-1 knockdown enhanced sensitivity to PTX and ADR by reducing P-glycoprotein expression through inhibiting the Raf-1/AP-1 signaling pathway **A**. The levels of P-gp, p-Raf-1 (Ser338), Raf-1, p-c-Jun (Ser73), c-Jun and c-Fos in MCF-7/PTX cells with Raf-1 siRNA1, Raf-1 siRNA2 or si-control transfection were detected by western blot. β-actin was used for normalization. **B**. The levels of P-gp, p-Raf-1 (Ser338), Raf-1, p-c-Jun (Ser73), c-Jun and c-Fos in MCF-7/ADR cells with GW5074 (5 and 10 μM) treatment were detected by western blot. β-actin was used for normalization. **C**. and **E**. With 15 nM PTX treatment, the relative cell viability and apoptosis in MCF-7/PTX cells cotransfected Raf-1 siRNA1 with pcDNA 3.1-MDR1 were assessed by MTT assay and flow cytometry. **D**. and **F**. With 300 nM ADR treatment, the relative cell viability and apoptosis in MCF-7/ADR cells with 10 μM GW5074 treatment and pcDNA 3.1-MDR1 transfection were assessed by MTT assay and flow cytometry. Data are presented as mean ± SD. *P < 0.05, ***P < 0.001.

The Raf-1 siRNAs and Raf-1 inhibitor GW5074 were used to inhibit Raf-1 activity to further observe the underlying mechanism of Gal-1 knockdown on MDR of breast cancer cells. The western blot results showed that Raf-1 siRNA1 and Raf-1 siRNA2 dramatically decreased the levels of P-gp, p-Raf-1 (Ser338), Raf-1, p-c-Jun (Ser73), c-Jun and c-Fos in MCF-7/PTX cells (Figure 5A), and Raf-1 inhibitor GW5074 obviously reduced the levels of P-gp, p-Raf-1 (Ser338), p-c-Jun (Ser73), c-Jun and c-Fos in MCF-7/ADR cells (Figure 5B), suggesting the validity of Raf-1 siRNAs and Raf-1 inhibitor GW5074 in suppressing the Raf-1/AP-1 signaling pathway.

The relative cell viability and apoptosis in MCF-7/PTX cells treated with (Raf-1 siRNA1 + PTX) or (Raf-1 siRNA1 + pcDNA-MDR1 + PTX) were assessed by MTT assay and flow cytometry. The results showed that combination of Raf-1 siRNA1 and PTX dramatically inhibited cell viability and induced cell apoptosis in MCF-7/PTX cells compared with (PTX + Con siRNA) group, whereas MDR1 overexpression abated these effects (Figure 5C and 5E). Additionally, MCF-7/ADR cells were treated with GW5074 or (GW5074 + ADR) or (GW5074 + pcDNA-MDR1 + ADR). MTT assay and flow cytometry showed that combination of GW5074 and ADR significantly decreased cell viability and promoted apoptosis in MCF-7/ADR cells compared with ADR treatment group, while MDR1 overexpression overturned these effects (Figure 5D and 5F). These data suggested that Galectin-1 knockdown enhanced sensitivity aganist PTX and ADR by decreasing P-gp expression through suppressing the Raf-1/AP-1 signaling pathway

## DISCUSSION

Accumulating evidence reveals that Gal-1, a member of the mammalian β-galactoside-binding proteins, is upregulated in different tumor types, such as breast cancer [19], lung cancer [20], and prostate cancer [21]. Gal-1 has distinct biological roles in tumor progression, including cell growth, apoptosis, metastasis, and immunosuppression and is involved in poor prognosis and the metastatic phenotype [22, 23]. For example, Zhang *et al*. reported that Gal-1 overexpression promoted tumorigenesis and Gal-1 knockdown led to the reduction in cell growth, migration and invasion in epithelial ovarian cancer (EOC) [24]. Zheng *et al*. showed that overexpression of Gal-1 promoted cell invasion and migration and suppressed cell apoptosis by enhancing TGF-β signaling in gastric cancer [25]. Wiest *et al*. manifested that Gal-1 showed apoptotic potential in human breast cancer and trophoblast tumor cells [26]. Zhu *et al*. reported that Gal-1 was highly expressed in human breast tumor tissues and Gal-1 knockdown in carcinoma-associated fibroblasts inhibited cell migration and invasion by suppressing the expression of matrix metalloprotein 9 (MMP-9) [19]. In the present study, we demonstrated that Gal-1 was highly expressed in breast tumor tissues and cells as compared with normal tissues and cells. Besides, Gal-1 expression was significantly higher in MDR breast cancer cells MCF-7/PTX and MCF-7/ADR. Gal-1 knockdown dramatically increased sensitivity to ADR and PTX in MCF-7/PTX and MCF-7/ADR cells by inhibiting cell proliferation and enhancing apoptosis.

MDR to chemotherapeutic agents is the main cause of chemotherapy failure of breast cancer. P-pg/MDR1 overexpression is one of the main causes leading to MDR phenomenon in breast cancer and plays a crucial role in producing MDR in breast cancer cells [27]. For example, He *et al*. reported that MDR1 silencing resulted in a decrease of P-pg activity and drug sensitivity of yolk sac carcinoma [28]. Liu *et al*. manifested that siMDR1 effectively reduced the expression of MDR1 and led to a remarkable increase of chemosensitivity to PTX [29]. In the present study, we discovered that the mRNA and protein expression levels of MDR1 were higher in breast tumor tissues and cells than that in normal breast tissues and cells and MDR1 overexpression obviously decreased sensitivity to ADR and PTX induced by Gal-1 knockdown in MCF-7/PTX and MCF-7/ADR cells by promoting cell proliferation and suppressing apoptosis.

Previous studies have reported that Raf and AP-1 signaling pathway is related to MDR [30]. For example, Mukherjee *et al*. reported that Her2/Raf-1/MAPK/AP-1 pathway contributed to the development of castrate-resistance prostate cancer, resulting in early relapse and reduced disease-specific survival [31]. Kreiseder *et al*. reported that the cytoskeletal linker protein α-catulin chemosensitized melanoma cells by activating NF-κB and AP-1 [32]. Yang *et al*. found that upregulation of miR-195 improved the sensitivity to ADR in breast cancer by suppressing Raf-1 [33]. In addition, it is convincingly demonstrated that both AP-1 and NF-κB were transcription factors of P-gp [34]. More importantly, NF-κB was demonstrated to induce drug resistance by improving the MDR1 gene expression in cancer cells [35]. Moreover, a previous study uncovered that ectopic expression of Gal-1 mediated chemoresistence of chronic myeloid leukemia cells by inducing MDR1 expression via P38 MAPK activation and NF-κB translocation [36]. In the present study, we noted that Gal-1 knockdown markedly reduced the levels of P-pg, p-Raf-1 (Ser338), p-c-Jun (Ser 73), c-Jun and c-Fos. These results manifested that Gal-1 knockdown enhanced sensitivity against PTX and ADR by reducing P-glycoprotein expression via suppressing the Raf-1/AP-1 signaling pathway

In conclusion, we found that Gal-1 and P-gp were both upregulated in breast tumor tissues and cells and Gal-1 knockdown dramatically improved drug sensitivity of breast cancer cells by reducing P-gp expression through inhibiting the Raf-1/AP-1 signaling pathway, providing a novel evidence that combining the depletion of Gal-1 with drugs PTX or ADR is a potential method for the therapy of patients with breast cancer.

## MATERIALS AND METHODS

### Cell lines and tumor tissues

Breast tumor tissues and corresponding normal tissues from 20 patients with breast cancer were collected from the First Affiliated Hospital of Zhengzhou University. The experiments were carried out with approval of the Ethic Committee of Zhengzhou University. Informed consent was obtained from all patients for using their tissues. The human breast cancer cell line (MCF-7) and human breast epithelial cell line (MCF-10A) were obtained from the American Type Culture Collection (ATCC, Manassas, VA, USA). MCR-7 derived adriamycin (ADR) resistant cells MCF-7/ADR and paclitaxel (PTX) resistant cells MCF-7/PTX were established as previously described [37], which were induced drug resistance by pre-treating MCF-7 cells with continuously increasing concentrations of ADR and PTX over 8 months. The MCF-7 and MCF-10A cells were grown in RPMI-1640 medium (Gibco-BRL, Carlsbad, CA, USA) containing 10% fetal bovine serum (FBS) (Gibco-BRL), 100 U/mL penicillin/streptomycin/kanamycin (Genom Co., Ltd., Hangzhou, China) at 37°C in a humidified atmosphere of 5% CO_2_.

### Cell transfection

Double strand siRNA nucleotides targeting Gal-1 (Gal-1 siRNA1 and Gal-1 siRNA2), double strand siRNA nucleotides targeting Raf-1 (Raf-1 siRNA1 and Raf-1 siRNA2), scrambled siRNA (Con siRNA), pcDNA 3.1-MDR1, and pcDNA 3.1 empty vectors were purchased from Shanghai GenePharma Co., Ltd (Shanghai, China). For transient transfection, 50,000 cells were seeded into six-well plates and incubated for 24 h at 37°C. Then the cells were transfected with different siRNAs, pcDNA-MDR1 or respective controls by Lipofectamine 2000 (Invitrogen, Grand Island, NY, USA) and incubated for 48 h in the serum-free medium. The cells with scrambled siRNA transfection were used as the control.

### Cell viability analysis

MCF, MCF-7/ADR, and MCF-7/PTX cells (2×10^4^ cells/well) were incubated in 96-well plates for 24 h and then the cells were transferred into 100 μl fresh culture medium containing different concentrations of PTX (2.5, 5, 10, 15, and 20 nM) or ADR (50, 100, 200, 300, and 400 nM). Cells with no additives were used as the control. Following 48 h incubation, 20 μl (0.5 mg/ml) MTT was added to each well and incubated for an additional 4 h at 37°C and then 200 μl DMSO was added to dissolve the MTT formazan precipitate for 15 min after the medium was removed. After the crystals were completely dissolved, the absorbance value at 490 nm in each well was analyzed by a microplate reader (SPECTRAmax; Molecular Device Corp., Sunnyvale, CA, USA). The IC50 values were calculated using the non-linear regression curve fit of the Graphpad Prism5 software. The cell viability in all transfected cells were detected using the method described above.

### Quantitative real-time PCR (qRT-PCR)

The RAN from the breast cancer cell lines, normal breast tissue and breast tumor tissue samples were isolated by TransZolTMUp (TransGen Biotech, Beijing, China). For the detection of Gal-1 and MDR1 mRNA expression, the complementary DNA (cDNA) was reversely transcribed using RT reagent kit (Takara, Shiga, Japan). The RT-PCR was used to evaluate the mRNA expression of Gal-1 and MDR1 with the SYBR green detection system (ThermoScientific, Waltham, MA, USA) in an ABI7500 sequence detection system (Life Technologies, Carlsbad, CA, USA). The procedure of RT-PCR was performed as follows: denaturing temperature 95°C for 30 s, 60°C for 30 sec, 35 cycles at 95°C for 30 s, and 60°C for 20 s. The primers used were as follows: forward, 5′-CTGTGCCTGCACTTCAACC-3′and reverse, 5′-CATCTGGCAGCTTGACGGT-3′ for *Gal-1*; forward, 5′-GGAGCCTACTTGGTGGCACATAA-3′ and reverse, 5′-TGGCATAGTCA GGAGCAAATGAAC-3′ for *MDR1*. The mRNA expression of GAPDH was used as the endogenous control.

### Western blot

For western blot analysis, the cells (1×10^6^/ml) with all siRNAs, pcDNA 3.1 MDR1 or respective controls transfection or with GW5074 (5 or 10 μM) (Sigma, St. Louis, MO, USA) treatment for 1 h were harvested and lysed in RIPA buffer (Beyotime Institute of Biotechnolgy, Haimen, China) for 30 min. The bicinchoninic acid (BCA) Protein Assay Kit (CoWin Biotechnology, Beijing, China) was used to detect the concentrations of proteins. Subsequently, samples (50 μg) were separated on 10% sodium dodecylsulfate-polyacrylamide gel electrophoresis (SDS-PAGE) (Beyotime Institute of Biotechnolgy) and then blotted onto PVDF membranes (Millipore, Billerica, MA,USA). Following blocked with 5% milk in Tris-buffer saline (TBST) at 37°C for 1 h, the membranes were incubated with monoclonal antibodies (MDR1/P-gp, p-Raf-1, Raf-1, p-c-Jun (Ser73), c-Jun, c-Fos, and β-actin) (Santa Cruz Biotechnology, Santa Cruz, CA, USA) with 1:500, 1:1000, 1:500, 1:1000, 1:500, 1:500, and 1:2000 dilutions overnight at 4°C, respectively. The membranes were then washed three times with TBST and labeled with horseradishperoxidase-conjugated anti-mouse secondary antibodies (1:5000 dilutions). Following washing three times, the blots were visualized by Electrochemiluminescence Plus Detection system (EMD Millipore, Billerica, MA, USA) and analyzed using an Image Scanner (Amersham Biosciences, Uppsala, Sweden). The β-actin was used as the internal control.

### Cell apoptosis assay

MCF-7/ADR and MCF-7/PTX cells transfected with Gal siRNA1, Gal siRNA2, si-control, pcDNA 3.1-MDR1, or pcDNA 3.1 empty vectors were seeded in six-well plates at a density of 2×10^5^ cells/well. Following incubation for 24 h, cells were treated with 15 nM PTX or 300 nM ADR for 48 h. Then the cells were collected and washed three times with PBS, followed by centrifugation at 2×10^3^ rpm for 5 min and resuspended in 100 μL binding buffer. Subsequently, 5 μL Annexin V-FITC was added for incubation for 10 min, followed by 5 μL propidium iodide (PI) for 15 min incubation. The apoptotic cells were analyzed by using the FACScan flow cytometry and CellQuest software (BD Biosciences, San Jose, CA, USA).

### Statistical analysis

Data from each group were presented as the mean ± standard deviation (SD) error from at least three independent experiments. Comparison between two groups was performed by Student's *t* test and differences for three or more groups were analyzed using one-way ANOVA tests. Statistical analysis was conducted using graph prism 5.0 software (GraphPad Prism, San Diego, CA). A statistically significant difference was considered at a *P* value less than 0.05.

## Abbreviations

Gal-1: Galectin-1
PTX: paclitaxel
ADR: adriamycin
MDR: multidrug resistance
ATP: adenosine triphosphate
P-gp: P-glycoprotein
MAPK: mitogen-activated protein
Con siRNA: control siRNA
EOC: epithelial ovarian cancer
MMP-9: matrix metalloprotein 9.
